# Preclinical study in a postoperative pain model to investigate the action of ketamine, lidocaine, and ascorbic acid in reversing fentanyl-induced, non–glutamate-dependent hyperalgesia

**DOI:** 10.1097/PR9.0000000000001062

**Published:** 2023-02-13

**Authors:** Marina Ayres Delgado, Luana Assis Ferreira, Bianka Jaciara dos Santos Gomes, Isis Katarine Orlandi Leite, Marcus Vinícius Gomez, Célio Castro-Junior

**Affiliations:** Programa de pós graduação em ciências da saúde, Santa Casa de Belo Horizonte Ensino e Pesquisa, Belo Horizonte, Minas Gerais, Brazil

**Keywords:** Ketamine, Lidocaine, Opioid-induced hyperalgesia, Thermal hypersensitivity, Mechanical allodynia

## Abstract

**Introduction:**

Opioid-induced hyperalgesia (OIH) is a paradoxical phenomenon in which exposure to opioids can increase sensitivity to painful stimuli. Currently, several drugs have been used in an attempt to prevent OIH. We design this study to address the effect of preemptive treatment with ketamine, lidocaine, and ascorbic acid in a rat preclinical model of perioperative opioid-induced hyperalgesia.

**Methods:**

To reproduce OIH in a model of postoperative pain, rats received successive doses of fentanyl subcutaneously and underwent an incision in the paw. In an attempt to prevent OIH, ketamine, lidocaine, and ascorbic acid were administered before treatment with fentanyl. The von Frey test and the hot-plate test were used to evaluate mechanical allodynia and thermal hyperalgesia, respectively, with a follow-up period from 1 hour up to 7 days after surgery. Spinal cord nerve terminals (synaptosomes) were used to assess glutamate release under our experimental conditions.

**Results:**

Consecutive fentanyl injections increased the postoperative pain as indicated by increased thermal hyperalgesia and allodynia 48 hours after incision. Ketamine, lidocaine, and the combination of ketamine + lidocaine were able to prevent thermal hyperalgesia but not mechanical allodynia. Ascorbic acid did not prevent the hyperalgesia induced by fentanyl. We found no correlation between spinal glutamate release and the pharmacological treatments.

**Conclusion:**

Fentanyl induced a hyperalgesic effect that last few days in a postoperative model of pain. Hyperalgesic effect was not totally inhibited by ketamine and lidocaine in rats. Increased glutamate release was not the main molecular mechanism of fentanyl-induced hyperalgesia.

## Abstract 2

Fentanyl induced a hyperalgesic effect and was not inhibited by ketamine, lidocaine, and ascorbic acid in rats. Observations show that large doses of opioids may increase postoperative pain.

## 1. Introduction

The management of acute postoperative pain remains a challenge.^21^ Opioids are the most commonly used analgesics in postoperative pain treatment; however, they can induce several adverse effects including dependence, constipation, respiratory depression, analgesic tolerance, and opioid-induced hyperalgesia.^21^ The concept of pain sensitization after opioid administration is referred to as opioid-induced hyperalgesia.^22^ Opioid-induced hyperalgesia is defined as an increase in nociceptive sensitization and perception of pain due to exposure to opioids. It is a paradoxical response related mainly to opioid agonists of µ receptors, and the principal features include hyperalgesia and allodynia, worsening of pain despite increasing doses of opioids, diffuse pain, and pain that extends beyond the local of the pre-existing pain.^17^ The opioid activation of pronociceptive systems may be responsible for the increased pain scores and analgesic requirements in the postoperative period.^5^ Several mechanisms have been proposed to elucidate opioid-induced hyperalgesia, including sensitization of the central glutaminergic system, increased expression of excitatory neuropeptides, increased spinal dynorphins, enhanced microglial reactivity, and individual genetic factors.^9,19^ Although opioid-induced hyperalgesia (OIH) and analgesic tolerance share some clinical features, they are different phenomena: increasing opioid dose aggravates pain in OIH, whereas tolerance does not.^13^ In this study, we tested the hypothesis that abrupt cessation of opioid administration at the time of hind paw incision in a rat model of incisional pain leads to thermal hyperalgesia and mechanical allodynia in the postoperative period, compared with naive animals. In addition to evaluating the development of fentanyl-induced hyperalgesia, we address the effects of the pretreatment with ketamine, lidocaine, ascorbic acid, and the combination of small doses of lidocaine and ketamine in the prevention of hyperalgesic state on those animals.

## 2. Material and methods

### 2.1. Animals

The study was approved by the ethics committee of animal experimentation of Santa Casa, Belo Horizonte, protocol number 002/2018. Fifty adult male Wistar rats weighing 180 to 250 g were used in the experiments. The animals were housed in groups of 5 per cage and maintained on a 12:12 h light–dark cycle at 22 ± 2°C. After weighing, the rats were acclimatized to minimize the influence of stress during the experimental procedure. The control group received the same volume of phosphate buffered saline (PBS), in identical conditions. The effective doses were based and adjusted on the metabolic rate of different species. The von Frey test and the hot-plate test are robust and well-established methods for assessing opioid analgesia, hyperalgesia, and nociceptive sensitivity in rodents; both tests are simple to conduct and allow each animal to function as its own matched control.

### 2.2. Drugs

Fentanyl citrate, ketamine hydrochloride, and ascorbic acid were administered subcutaneously. Lidocaine and the combination lidocaine + ketamine were administered intravenously and were dissolved in PBS. Control animals received an equal volume of PBS injections. Doses of ketamine, lidocaine, their combination, and ascorbic acid were based on previous studies.^3–20^ The behavioral tests were performed by an experimenter who was blinded to the treatment animals received.

### 2.3. Von Frey tests

The hind paw withdrawal response to von Frey filament stimulation was evaluated. Each animal was placed in a wire cage through which von Frey filaments were applied. The animals were placed in the cage 30 minutes before the test for acclimatization. Eight von Frey filaments were used, and the binding force ranged from 1 to 60 g. The filaments, from the thinnest to the thickest, were applied with a stimulus interval of 5 seconds. The lowest force that produced a withdrawal response was considered the threshold. The stress response to the various filaments producing different degrees of mechanical stimulation (harmless or noxious) was analyzed. Sessions in rats began with the application of the 6-g filament. If the stress response was harmful, a lower value (g) filament was used from the last response. Each stimulation was spaced from the other stimulations in 30 seconds. Von Frey filaments were applied for 6 sessions, and paw withdrawal was recorded as a percentage of responses. The formula used to assess the 50% threshold in the mechanical allodynia model was 50% threshold = last wire log + (k × average), where last wire log = the value of the last filament used in the series of 6 applications of von Frey filaments, K = constant based on the Dixon table; mean differences in log filament strength for rats.

### 2.4. Hot-plate test

Thermal hyperalgesia was assessed using a hot-plate apparatus (Insight). The hot-plate test was always performed after von Frey tests. The animals were placed one at a time on a hot plate maintained at a temperature of 52°C. Latency of response by either jump or hind paw lick was recorded using an electronic timer. To prevent tissue damage, a cut-off time of 20 seconds was adopted. Each rat was measured 3 times, with at least 10-minute intervals between each measurement, and the average value was calculated.

### 2.5. Open field test

To assess whether the effect on exploratory and locomotor activity is negative after administration of the drugs, the animals were placed in photocells of the VersaMax device and the photocells have a dimension of 40 × 12 × 40 cm. Motor activity was measured using an activity monitor connected to the VersaMax equipment, using 3 infrared light detectors, each located in a photocell. The parameters analyzed were horizontal activity, vertical activity, total distance covered (cm), number of movements, time of movements (seconds), movement in the center (duration in seconds that the animal kept moving in the center of the box), and rearing (number of times the animal stands up and stands on both hind legs).

### 2.6. Fentanyl-induced hyperalgesia

The animals were randomly assigned to receive fentanyl or PBS through a subcutaneous route. We first studied the effectiveness of fentanyl in induced pain sensitization in the presence and the absence of incisional pain. The experiments were performed using 5 animals per group. Initially, to induce OIH, fentanyl was injected subcutaneously with a 25-gauge needle. To mimic the high-dose opioid treatment used in human surgeries, fentanyl was injected 4 times (60 µg/kg per injection) at 15-minute intervals, resulting in a total dose of 240 µg/kg administered subcutaneously (s.c). over 1 hour to induce hyperalgesia. This model is according to Celerier et al.^4^ Surgery was performed after isoflurane anesthesia, just after the fourth fentanyl injection. The model of incisional pain was originally described by Brennan et al.^23^ After antisepsis of the animal's right hind paw with alcoholic chlorhexidine, a longitudinal incision (∼1 cm) was made on the plantar surface of the paw with the animal under inhalation isoflurane (2.5%). The incision was started immediately distal to the heel and extended to a point just proximal to the first set of footpads. After hemostasis was obtained, three 4-0 nylon sutures were placed along the wound. An inhalational anesthesia system consisting of an uncalibrated vaporizer, an induction chamber, and a gas source was used, keeping the animal comfortable and pain-free. A heating pad was provided during the procedure. The rats regained consciousness approximately 2 minutes after the surgery. Nociception was evaluated using the von Frey test and hot-plate test on the first and fourth hours after the surgery and then on days 1, 2, 3, 4, and 7 after the surgical procedure.

### 2.7. Effects of ketamine, ascorbic acid, lidocaine, and the combination of small doses of lidocaine + ketamine on fentanyl enhancement of pain behavior induced by surgery

We studied the role of ketamine, lidocaine, and ascorbic acid in preventing opioid-induced sensitization to surgical pain. Thirty-six rats were separated into 6 groups. In all groups, fentanyl 60 µg was injected 4 times s.c. at 15-minute intervals, resulting in total doses of 240 µg. In the control group, PBS was injected s.c. 15 minutes before fentanyl. In the ketamine group, ketamine 10 mg/kg was injected s.c. 15 minutes before fentanyl. In the lidocaine group, lidocaine 5 mg/kg was injected intravenously 15 minutes before fentanyl. In the combination group, the combination of lidocaine and ketamine (0.34 mg/kg + 0.76 mg/kg) was injected intravenously 15 minutes before fentanyl. In the ascorbic acid group, ascorbic acid 5 mg/kg was injected s.c. 15 minutes before fentanyl. Surgery was performed as described above after the last injection. Testing (the von Frey test and the hot-plate test) was performed before the beginning of the experiment to collect baseline values and then 1 and 4 hours after surgery and once daily during the first 7 postoperative days after the surgery. Experiments were conducted following a double-blind protocol.

### 2.8. Synaptosomal assay

Synaptosomes were prepared from the spinal cord of male Wistar rats. The rats were decapitated, and the spinal cord was quickly removed and placed in aerated ice-cold–modified Krebs-Ringer buffer with minor modifications. The spinal cord was dissected from the rats and homogenized in 14 mL of ice-cold 0.32 M sucrose. The crude synaptosomal pellet (P2) was prepared by differential centrifugation with 1000 g for 5 minutes, followed by 15,000 g for 20 minutes. Protein was assayed by the Bradford method. In all synaptosomal experiments, the P2 pellet was resuspended in 4.5 mL of Krebs-Ringer buffer, aerated with 95% O_2_/5% CO_2_. For the test, 500 µL of the synaptosomal suspension was diluted with 1500 µL of Krebs buffer containing 5 µL of NADP+ (1 nM) and glutamate dehydrogenase (50U). This assay relies on the generation of nicotinamide adenine dinucleotide phospate (NADPH) by glutamate dehydrogenase in the presence of glutamate, with NADPH being measured fluorometrically. The excitation wavelength was set at 360 nm and the emission wavelength to 450 nm in a spectrofluorometer. Wistar male rats treated with fentanyl were decapitated and spinal cords were prepared in a cutting solution containing ketamine, lidocaine, and ascorbic acid. The experiment aimed to demonstrate the role of glutamate in fentanyl-induced hyperalgesia and the efficacy of ketamine, lidocaine, and ascorbic acid in the prevention of this hyperalgesia.

### 2.9. Statistical analysis

The results are expressed as a percentage of the overall decrease of the nociceptive threshold, taking the PBS group as control. *P* < 0.05 was considered significant. The data presented in this work are expressed as mean ± standard error. Data were analyzed by one-way or 2-way analysis of variance (ANOVA), followed by the Dunnett multiple comparison test. GraphPad 7 was used as software for creating graphs and statistical analysis. For the detection of outliers in the sample, the QuickCalcs calculator provided by GraphPad was used, which performs the Grubbs test, also called the ESD (extreme studentized deviate) method, determining whether one of the sample values is a value significantly external from the rest.

## 3. Results

### 3.1. Opioid-induced hyperalgesia

**Figure 1.**
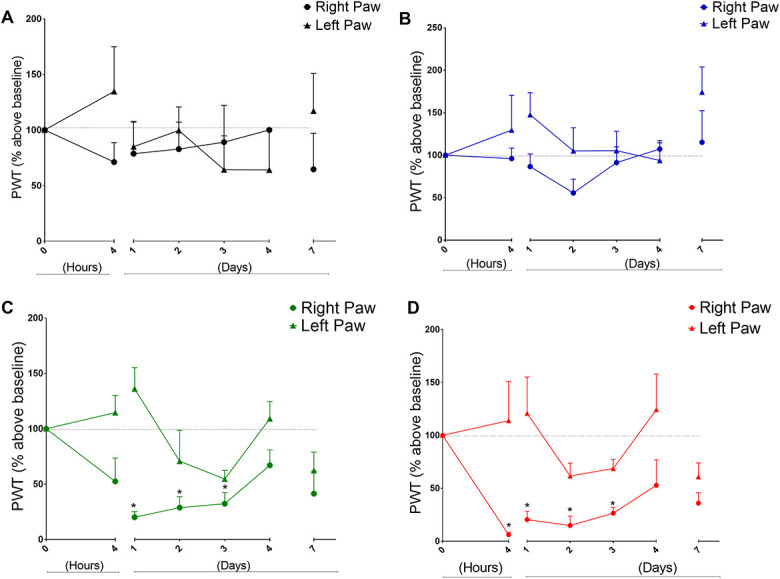
Time curves of mechanical nociceptive thresholds under different experimental conditions. In all graphs, time 0 represents basal paw withdrawal threshold (PWT). Consecutive measurements of the PWT were performed as indicated by the *x*-axis. (A) Control group of animals that received 4 consecutive injections of PBS only. (B) After basal PWT measurements, animal received 4 consecutive doses of fentanyl every 15 minutes (60 μg/kg each, 240 μg/kg total). (C) Animals received fentanyl (4 × 60 μg/kg) and underwent surgery in the right hind paw. (D) Animals received PBS (without fentanyl) and underwent surgery. Points represents mean ± SEM (*P < 0.05, ANOVA with repeated measures, N = 5–8 animals per group). ANOVA, analysis of variance.

Administration of 4 consecutive doses of fentanyl (60 µg/kg each injection, 240 µg/kg in total) induced a slight although not statistically significant decrease in the mechanical nociceptive threshold (PWT) of the rats (Fig. 1B). However, animals that received fentanyl and underwent surgery on the right hind paw (Fig. 1C), similarly to animals that underwent surgery without fentanyl (Fig. 1D), showed a remarkable ipsilateral drop in the mechanical thresholds that reach statistical significance from day 1 up to day 3. Of note, complementary comparisons of PWT between fentanyl + surgery vs surgery (only) groups showed no statistical significance in any of the time points tested (2-way ANOVA with Dunnett posttest).

**Figure 2.**
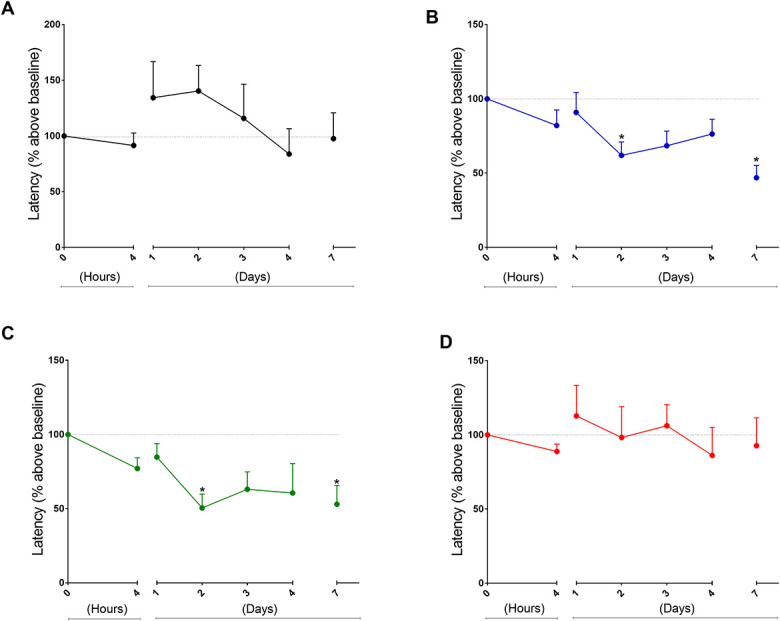
Time curves of thermal nociceptive latency (hot-plate test) under different experimental conditions. In all graphs, time 0 represents normalized basal latency. Consecutive measurements of the latencies were performed as indicated by the *x*-axis. (A) Control group of animals that received 4 consecutive injections of PBS only. (B) After basal PWT measurements, animal received 4 consecutive doses of fentanyl every 15 minutes (60 μg/kg each, 240 μg/kg total). (C) Animals received fentanyl (4 × 60 μg/kg) and underwent surgery in the right hind paw. (D) Animals received PBS (without fentanyl) and underwent surgery. Points represents mean ± SEM (*P < 0.05, ANOVA with repeated measures, N = 5–8 animals per group). ANOVA, analysis of variance.

Data from experiments addressing the thermal nociceptive thresholds are presented in Figure 2. Right after von Frey tests, animals were harvested in the hot plate at similar time points as it was shown for the mechanical threshold data. Consecutive injections of fentanyl produced a significant drop in the thermal nociceptive threshold (Fig. 2B). Similar drops in the thermal threshold have been observed in the fentanyl + surgery group (Fig. 2C) but not on animals that underwent surgery without fentanyl (surgery only group, Fig. 2D). Complementary analysis revealed significant differences between these 2 groups at day 2 and day 7 (2-way ANOVA with Dunnett posttest). Together, these data show—under our experimental conditions—that thermal hyperalgesia but not mechanical hyperalgesia was more pronounced in the fentanyl + surgery group suggesting thus fentanyl enhanced the magnitude of the thermal nociception after surgery.

### 3.2. Effect of ketamine, lidocaine, and ascorbic acid on hyperalgesia induced by fentanyl

**Figure 3.**
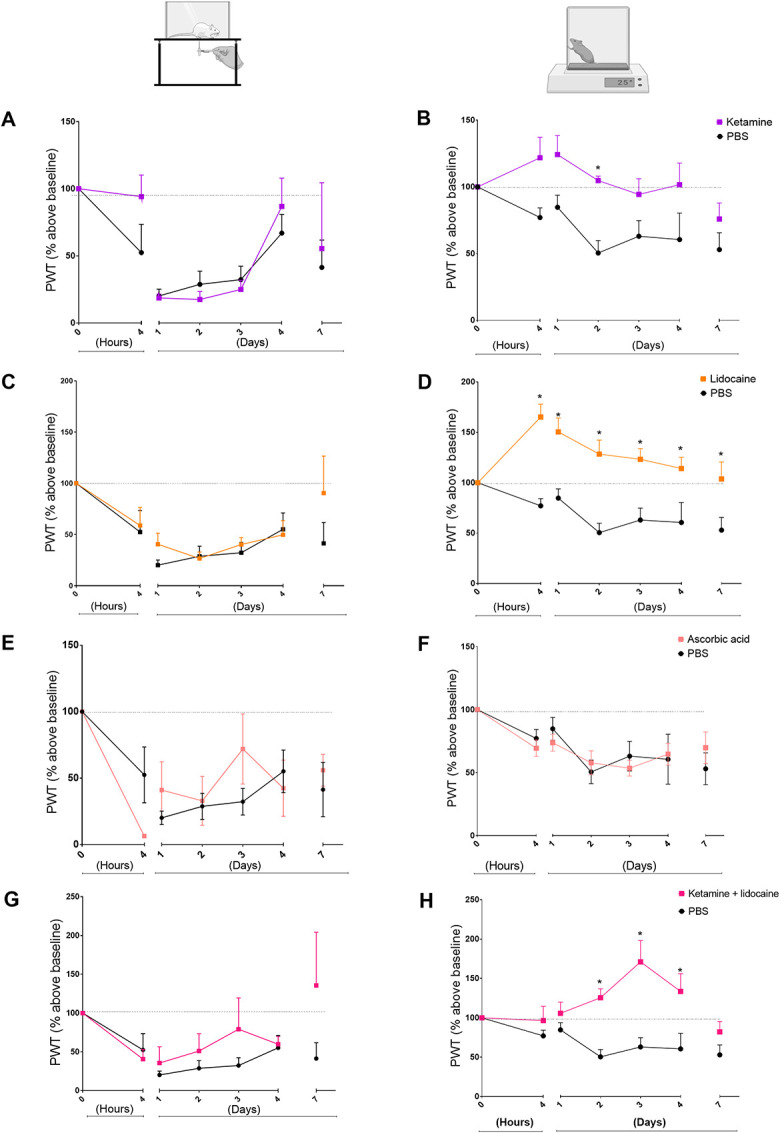
Effects of ketamine, lidocaine, combination of ketamine and lidocaine, and ascorbic acid in the prevention of fentanyl-induced hyperalgesia. Time 0 in all graphs indicates the measurements taken before the animals underwent surgery and fentanyl injection. Threshold values are presented as % of baseline and were normalized based on the threshold at time 0. All animals underwent fentanyl OIH induction and surgical procedure on the right hind paw. Graphs on the left side display paw withdrawal threshold values in seconds in the hot-plate test, and graphs on the right side displays right hind paw withdrawal threshold values in the test in grams obtained through the von Frey test. In all graphs, the black line represents the control group of animals and the colorful lines represent animals receiving ketamine (A and B), lidocaine (C and D), ascorbic acid (E and F), and the combination of lidocaine and ketamine (G and H) as a preventive treatment for OIH. *P < 0.05 compared with the PBS group. Two-way ANOVA, followed by the Dunnett multiple comparison test, N = 5–8 animals per group. ANOVA, analysis of variance; OIH, opioid-induced hyperalgesia.

Fentanyl effects on mechanical nociceptive threshold were not prevented by previous ketamine administration and no reduction in mechanical nociceptive threshold was observed during the entire duration of the experiment. However, ketamine prevented the thermal hyperalgesia induced by fentanyl (Figs. 3A, B). Intravenous lidocaine in doses of 5 mg/kg do not effectively prevent the mechanical allodynia induced by fentanyl. Lidocaine prevents fentanyl-induced thermal hyperalgesia. The thresholds of animals receiving lidocaine are higher with a significant difference compared with the fentanyl group in the hot-plate test. Regarding the von Frey test, animals that receive lidocaine + fentanyl do not present thresholds significantly different from the thresholds of the fentanyl group (Figs. 3C, D). Subcutaneous ascorbic acid in doses of 5 mg/kg do not prevent the hyperalgesic state induced by fentanyl in the behavioral tests (Figs. 3E, F). Intravenous combination of ketamine and lidocaine in the doses of 0.34 mg/kg and 0.76 mg/kg presented higher paw withdrawal thresholds with a significant difference when compared with the fentanyl group in the thermal nociceptive test. In the von Frey test, animals that receive the combination do not present thresholds significantly different from the thresholds of the PBS group at any time observed. However, at day 3, the thresholds were higher although without statistical significance. The combination seems to improve the sensitivity at the end of the experiment (Figs. 3G, H).

**Figure 4.**
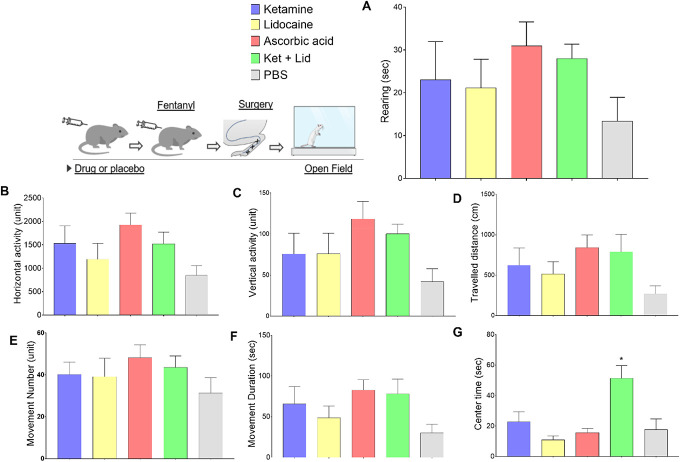
Absence of side effects related to the drugs in spontaneous locomotion on an open field test. All groups in this trial received 240 µg/kg fentanyl to establish a model of OIH. The groups differ in the type of drug chosen for OIH prevention. The group receiving ketamine is represented by the blue bar. The group receiving lidocaine is represented by the yellow bar. The group receiving ascorbic acid is represented by the red bar. The group receiving ketamine + lidocaine is represented by the green bar. The group that receives PBS is represented by the gray bar. The bars are in that order. *P < 0.05 compared with the PBS group. ANOVA followed by the Dunnett multiple comparisons test, N = 5–8 animals per group. ANOVA, analysis of variance; OIH, opioid-induced hyperalgesia.

Spontaneous motor activity was evaluated 2 hours after the surgical procedure and the data are presented in Figure 4. The following parameters were recorded: rearing (Fig 4-A), horizontal activity (Fig 4-B), vertical activity in cm (fig 4-C), distance covered (fig 4-D), number of movements (fig 4-E), duration of movements in seconds (fig 4-F), and time of movements in the center (4-G). No changes were observed in the behavior of the animal in the open field after 2 hours of the surgical procedure (approximately 3 hours after drug administration). For the parameter “movement time” in the center of the box, we observed a significant difference between the group that receives combination of ketamine and lidocaine vs the PBS group (*P* = 0.0036).

### 3.3. Effect of fentanyl in glutamate levels in the spinal cord

**Figure 5.**
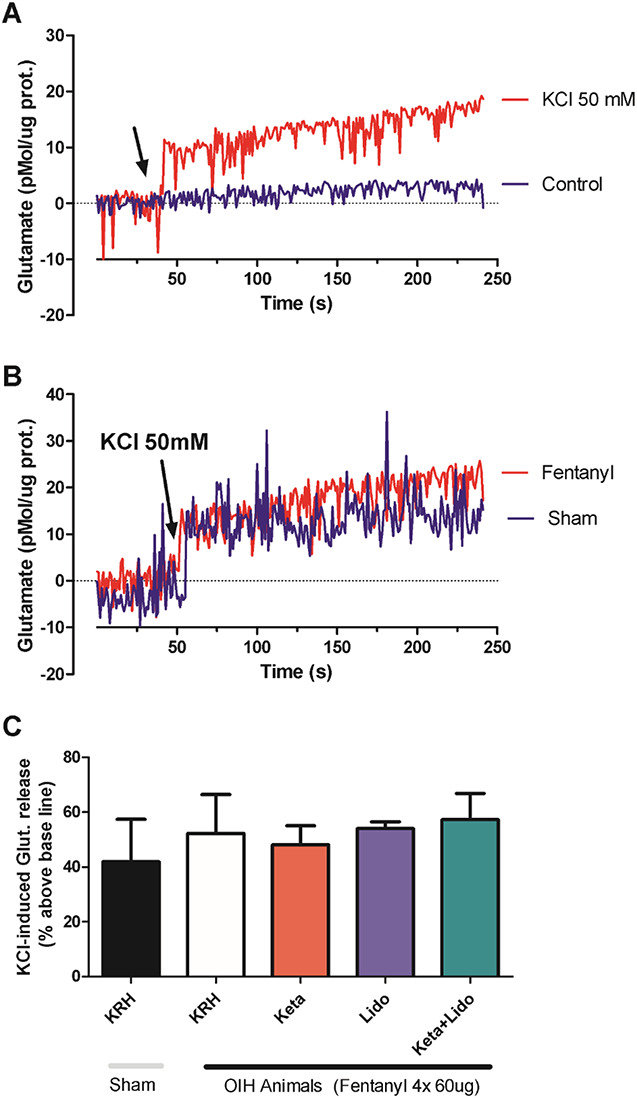
Absence of effect of OIH and treatments on glutamate release in spinal cord synaptosomes. (A) Glutamate levels are expressed as a function of time. The arrow indicates the timing of the addition of 50 mM potassium chloride (KCl) to the synaptosomal preparation (red line) or the addition of the corresponding volume of physiological solution (krebs Ringer hepes or physiological solution [KRH], blue line). The KCl-mediated depolarizing stimulus triggers glutamate release from synaptosomes indicated by increased fluorescence levels. (B) KCl-induced depolarization triggers a similar elevation in glutamate levels in synaptosomes from animals previously treated with physiological solution (sham group, blue line) or animals treated with 4 doses of 60 µg fentanyl (fentanyl group, red line). (C) Quantification, in bars, of the KCl-induced glutamate elevation in the different experimental conditions as indicated. Incubation with ketamine, lidocaine, or ketamine + lidocaine was performed directly on the synaptosomal preparation 5 minutes before the addition of KCl. (N = 5 independent experiments; *P* > 0.05 1-way ANOVA). ANOVA, analysis of variance; OIH, opioid-induced hyperalgesia.

Glutamate released in the spinal cord at the first-order synapse of the ascending nociceptive pathway is an important mediator of pain, including postoperative pain. Fentanyl injected into the animals did not modify the KCl-induced glutamate levels in the spinal cord synaptosomes. Data shown in Figure 5 **(A-G)** demonstrate the release of glutamate from synaptosomes obtained by homogenizing the spinal cord of fentanyl-treated rats compared with synaptosomes from PBS-treated rats. There was no statistically significant difference between the groups. Preincubation of the synaptosome with ketamine, lidocaine, and the combination of lidocaine and ketamine did not modify spinal cord glutamate levels as well.

## 4. Discussion

Opioids are generally administered during anesthesia to promote analgesia and reduce anesthetics requirements.^1^ Hyperalgesia may develop in association with opioids.^1^ The possibility that opioid therapy may sensitize pronociceptive pathways and may impair the treatment of pain or even aggravate pre-existing pain has to be considered, mainly when high opioid doses are used. Opioid-induced hyperalgesia should be suspected when the opioid effect seems to wane in the absence of disease progression, unexplained pain reports, or diffuse allodynia unassociated with the site of injury.^6^ The present study was designed to explore the influence of acute administration of the opioid agonist fentanyl on the thermal hyperalgesia and mechanical allodynia observed after hind paw incision in rats. Celerier et al.^4^ demonstrated that fentanyl is able to induce hyperalgesia after acute systemic administration in rats and also in uninjured humans. In addition, based on the study by Celerier, we defined a sample size of 5 to 8. Overall, this is not a large sample size and represents the main limitation of the study. According to Mauermann et al.,^16^ a higher dose of fentanyl increased hyperalgesia from 4.5 to 6.5 hours in healthy volunteers after intracutaneous electrical stimulation. Our data are consistent with the related hypothesis that acute administration of opioids can alter thermal hyperalgesia and mechanical allodynia after hind paw incision. Because the pronociceptive effect of opioids has been shown to bare dose related, we used high doses of fentanyl. According to DeYong Liang,^14^ postincisional pain has been observed to be more severe in patients who chronically consume opioids. Postoperative pain management is a clinical challenge that can be complicated by opioid-induced hyperalgesia. Hyperalgesia after cessation of opioids was mentioned in many studies.^8,12,13^ As recovery from the surgical insult occurs, the effect of opioid-induced hyperalgesia becomes evident. In a rat model of postoperative pain, it has been demonstrated that fentanyl-induced pain sensitization is additive with the hyperalgesia and allodynia resulting from surgical injury.^4,5^ When fentanyl is given during surgery as part of the general anesthesia, it can significantly enhance and prolonged incisional pain in the postoperative period and can possibly play an important role in the chronification of pain after surgery. Although the precise molecular mechanisms of OIH are not yet understood, the central glutaminergic system is considered the most common possibility.^11^ This study sought to analyze what would be the preventive action of drugs with the translational potential of treating OIH. The results showed that the drugs ketamine, lidocaine, and ascorbic acid were not 100% effective in preventing the hyperalgesia that was induced by fentanyl together with the postoperative pain model. The present work also analyzed the action of the combination of lidocaine and ketamine in this same model of pain. The combination of 2 drugs can be effective in several types of treatment; however, this combination was also not 100% effective in preventing hyperalgesia. One possibility to explain the lack of effectiveness of ketamine in preventing OIH is the administration in a single bolus dose, which probably did not promote sufficient blockade of NMDA **N-methyl-D-aspartate** receptors.^7^

The limited ability of ketamine to avoid OIH provides evidence that NMDA receptors is not the main mechanism involved in hyperalgesic states.^8^

Ascorbic acid is a water-soluble antioxidant vitamin and is believed to act as a neuromodulator. It also seems to modulate glutamatergic transmission and blocks the NMDA receptors through altering the redox changes on the NMDA receptor.^10–20^ Hung et al.^10^ attributed the antinociceptive effect of ascorbic acid to its antioxidant and neuromodulatory properties and its role as a free radical scavenger. However, in this work, the acute and subcutaneous administration of ascorbic acid was not able to inhibit mechanical allodynia and thermal hyperalgesia produced by fentanyl. This failure of ascorbic acid action can be related to insufficient serum levels of the drug after administration of only 1 dose and by the chosen route, the subcutaneous route, which requires less technique to be administered and is widely used in an animal model. These findings are in agreement with the study by Saffarpour and Nasirinezhad^20^ where relief of neuropathic pain occurred only after the second week of chronic treatment with ascorbic acid.

It seems that a minimal pharmacological concentration is necessary for the beneficial effects on pain relief, immune function, antioxidant capacity, and endothelial function of ascorbic acid.

The beneficial effects of the intravenous lidocaine have been demonstrated in abdominal surgeries but remain controversial in other types of surgeries. The continuous infusion of lidocaine in the perioperative period is safe because the lidocaine's therapeutic index remains very high and the plasma concentrations stay largely below the cardiotoxic and neurotoxic threshold levels.^3^ As inflammation produced by glial cells plays a role in the generation of central sensitization and OIH, our data indicate that intravenous lidocaine may alleviate the inflammation caused by the activation of glial cells in the spinal dorsal horn. According to Ma et al., intravenous lidocaine may inhibit the activation of microglia and astrocytes in the spinal dorsal horn and downregulates the expression of TNF-alfa and IL-1 beta.^15^ Beaussier et al. demonstrated that intravenous lidocaine was able to reduce hyperalgesia caused by the surgical incision through peripheral and central mechanisms.^3^ However, in this study, lidocaine was not able to inhibit fentanyl-induced hyperalgesia at all times, but it was effective at separate points that should be further evaluated. In the hot-plate test, eg, lidocaine was effective in preventing thermal hyperalgesia after surgery. The same does not happen in the von Frey test. Perhaps the lack of efficacy at all times and in both tests is due to the dose of lidocaine used as a bolus and not as a continuous infusion, as used in previous studies. No studies were found in the literature evaluating the antinociceptive properties of the combination of ketamine and lidocaine. The combination of equieffective doses of ketamine and lidocaine may result in a dose-dependent synergistic antinociceptive interaction. Several possible mechanisms may explain synergism between drug pairs: one drug increases the affinity of the other for the receptor, one drug increases the availability of the other at the receptor, and one drug reduces the elimination of the other. Thus, the synergism between lidocaine and ketamine can be explained by the action of these drugs on various receptors related to pain. In this study, the combination of lidocaine with intravenously administered ketamine was able to prevent fentanyl-induced thermal hyperalgesia at some observed times; however, regarding mechanical allodynia, the combination did not show any benefit in preventing hyperalgesia.

One of the molecular mechanisms considered to be the most important in the development of OIH is the increase in glutamate release with consequent increase in NMDA receptor activity. In agreement with that, we evaluate the glutamate release in the synaptosomes of spinal cord of Wistar rats treated with high doses of fentanyl. Fentanyl did not increase the release of glutamate in the synaptosomal experiment. Thus, probably other more important mechanisms must be involved in the development of fentanyl-induced hyperalgesia. It should also be mentioned that perhaps glutamate may play an important role in hyperalgesia induced by other opioids, such as remifentanil. This is an important finding because the available analgesic drugs have a limited therapeutic value in the management of OIH. Araldi et al.^2^ showed that fentanyl induces acute hyperalgesia mediated by the release of Ca^2+^ from the endoplasmic reticulum through the ryanodine receptor. Given the complexity of the signaling pathway, the administration of a calcium blocker, such as dantrolene, or a ligand of the α2δ subunit of voltage-dependent calcium channels (which attenuates calcium channels influx), such as gabapentin, combined or not with lidocaine could be a therapeutic option in the prevention of fentanyl-induced hyperalgesia. In conclusion, fentanyl induced an analgesic effect and an opposite hyperalgesic effect that last few days. Hyperalgesic effect was not totally inhibited by ketamine, lidocaine, and ascorbic acid in rats. Our study corroborates clinical observations showing that relatively large doses of opioids may increase postoperative pain and morphine consumption.

## Disclosures

The authors have no conflict of interest to declare.
